# RNA-Seq Approach for Genetic Improvement of Meat Quality in Pig and Evolutionary Insight into the Substrate Specificity of Animal Carbonyl Reductases

**DOI:** 10.1371/journal.pone.0042198

**Published:** 2012-09-04

**Authors:** Won Yong Jung, Seul Gi Kwon, Minky Son, Eun Seok Cho, Yuno Lee, Jae Hwan Kim, Byeong-Woo Kim, Da Hye Park, Jung Hye Hwang, Tae Wan Kim, Hwa Choon Park, Beom Young Park, Jong-Soon Choi, Kwang Keun Cho, Ki Hwa Chung, Young Min Song, Il Suk Kim, Sang Keun Jin, Doo Hwan Kim, Seung-Won Lee, Keun Woo Lee, Woo Young Bang, Chul Wook Kim

**Affiliations:** 1 Department of Animal Resources Technology, Gyeongnam National University of Science and Technology, Jinju, Korea; 2 Swine Science and Technology Center, Gyeongnam National University of Science and Technology, Jinju, Korea; 3 SeqGenesis, Daejeon, Korea; 4 Division of Applied Life Science (BK21 Program), Gyeongsang National University, Jinju, Korea; 5 Plant Molecular Biology and Biotechnology Research Center (PMBBRC), Gyeongsang National University, Jinju, Korea; 6 Da-San-Jong-Don Co. Ltd., Namwon, Korea; 7 National Institute of Animal Science, RDA, Suwon, Korea; 8 Division of Life Science, Korea Basic Science Institute, Daejeon, Korea; 9 Graduate School of Analytical Science and Technology, Chungnam National University, Daejeon, Korea; University of Queensland, Australia

## Abstract

Changes in meat quality traits are strongly associated with alterations in postmortem metabolism which depend on genetic variations, especially nonsynonymous single nucleotide variations (nsSNVs) having critical effects on protein structure and function. To selectively identify metabolism-related nsSNVs, next-generation transcriptome sequencing (RNA-Seq) was carried out using RNAs from porcine liver, which contains a diverse range of metabolic enzymes. The multiplex SNV genotyping analysis showed that various metabolism-related genes had different nsSNV alleles. Moreover, many nsSNVs were significantly associated with multiple meat quality traits. Particularly, *ch7:g.22112616A>G* SNV was identified to create a single amino acid change (Thr/Ala) at the 145th residue of H1.3-like protein, very close to the putative 147th threonine phosphorylation site, suggesting that the nsSNV may affect multiple meat quality traits by affecting the epigenetic regulation of postmortem metabolism-related gene expression. Besides, one nonsynonymous variation, probably generated by gene duplication, led to a stop signal in porcine testicular carbonyl reductase (PTCR), resulting in a C-terminal (E281-A288) deletion. Molecular docking and energy minimization calculations indicated that the binding affinity of wild-type PTCR to 5α-DHT, a C_21_-steroid, was superior to that of C-terminal-deleted PTCR or human carbonyl reductase, which was very consistent with experimental data, reported previously. Furthermore, P284 was identified as an important residue mediating the specific interaction between PTCR and 5α-DHT, and phylogenetic analysis showed that P284 is an evolutionarily conserved residue among animal carbonyl reductases, which suggests that the C-terminal tails of these reductases may have evolved under evolutionary pressure to increase the substrate specificity for C_21_-steroids and facilitate metabolic adaptation. Altogether, our RNA-Seq revealed that selective nsSNVs were associated with meat quality traits that could be useful for successful marker-assisted selection in pigs and also represents a useful resource to enhance understanding of protein folding, substrate specificity, and the evolution of enzymes such as carbonyl reductase.

## Introduction

The pig, a major source of meat-based protein, is an economically important livestock animal. For the sake of improving genetic traits of economic importance, pigs have been bred using artificial selection. Among such traits, meat quality traits in particular have been included in genetic improvement programs because mechanisms controlling the traits are strongly associated with various internal intrinsic factors such as meat color, firmness, wetness, and intramuscular fat contents that are primarily determined by the postmortem metabolism, regulated by the altered functionality of native proteins during conversion of muscle to meat [1], [2], which is in turn affected by the coordinated inheritance of genetic variations [3], [4]. Accordingly, genetic variations have been an important resource for marker-assisted selection of genetic markers for pork meat quality traits.

Single nucleotide variations (SNVs) especially can affect meat quality development by altering or disrupting various metabolic pathways depending on where the variations are located in the genome, such as in regions encoding protein-coding or noncoding regions. For example, variations in non-coding regions often affect gene splicing, CpG methylation, transcription factor binding, or the sequence of noncoding RNA, leading to altered levels of gene expression [5], [6], [7], [8], [9]. Noticeably, variations in protein-coding regions give rise to nonsynonymous change in the amino acid sequence of the encoded protein and thus, are more likely to affect the protein structure and function [10], [11], probably leading to the critical effects on a phenotype of interest such as meat quality trait [12]. In addition, it has been estimated that 20–30% of nsSNVs affect protein function [13], [14]. Accordingly, nonsynonymous single nucleotide variation (nsSNV) has been a subject of recent interest in studies of the protein structure and function [10], [11] and even in genetic improvement of a phenotype in human and livestock [15], [16], [17], [18]. Therefore, the abundance of nsSNV data can facilitate the identification of protein folding changes through structural and phylogenetic comparisons and reveal evolutionarily conserved sites that may be critical for protein function. Furthermore, structural and functional information can provide important insights into associations between nsSNVs and meat quality traits.

To obtain massively unbiased information on nsSNVs, whole-genome resequencing is a useful method [19], [20], but it is very expensive and time consuming. Recently, there have been several examples of next-generation transcriptome sequencing (RNA-Seq) for nsSNV discovery. The RNA-Seq technique was first conducted by Chepelev et al. to identify SNVs in expressed exons of the human genome [21] and, thereafter, was applied to the discovery of SNVs in the bovine milk transcriptome and subjected to further validation by genotyping analysis [22]. From both of these studies, RNA-Seq was also demonstrated to be a cost-effective and time-saving strategy for the systematic identification of nsSNVs in the expressed regions of the genome. Moreover, the strategy is capable of significantly increasing the efficiency of selectively identifying specific trait-related nsSNVs when RNA samples from a specific trait-related tissue or cell are used for RNA-Seq [21], [22]. Thus, this may be a reasonable method for the selective discovery of porcine nsSNVs that affect meat quality-related metabolism, despite the lack of a complete genome sequence for the pig.

In this study, porcine RNA-Seq analysis was carried out to discover nsSNVs significantly associated with pork meat quality traits. For this, porcine liver RNA samples were chosen to selectively identify metabolism-related nsSNVs, since liver tissue produces diverse metabolic enzymes. Using this approach, several nsSNV candidates were identified, and, among these, a few candidates were validated by genotyping analysis. Furthermore, it was assessed whether or not they were significantly associated with meat quality traits. In addition, this approach, which integrates structural and phylogenetic analyses, was applied to porcine testicular carbonyl reductase (PTCR), which catalysis the NADPH-dependent reduction of aldehydes or ketones on a large number of carbonyl compounds, including steroid hormones [23].

Taken collectively, our approach integrating RNA-Seq and genotyping analyses revealed that selective nsSNVs are associated with meat quality traits and that they may be useful for successful marker-assisted selection in pigs. The RNA-Seq approach should also provide a useful resource for understanding protein folding, substrate specificity, and enzyme evolution, when combined with structural and phylogenetic approaches.

## Results and Discussion

### Identification of the porcine liver transcriptome by RNA-Seq

To discover nsSNVs related to postmortem metabolism, transcriptome sequencing (RNA-Seq) was performed using total RNA from liver tissues of four genetically different porcine breeds, *Berkshire*, *Duroc*, *Landrace,* and *Yorkshire*. The analysis included a total of 97,524,874 trimmed reads, and an average of 24,381,219 reads was obtained for each porcine sample. The trimmed reads were further assembled and mapped to UniGene, an annotated pig transcriptome assembly. According to the mapping results, among the total trimmed reads, 52,417,707 reads, ∼54% as a total mapping percentage, were categorized as mapped reads, corresponding to exon reads. Next, RPKM (reads per kilobase per million mapped reads) values (Mortazavi et al. 2008) were used to identify the total number of genes expressed in the porcine liver. Using RPKM threshold values greater than 1, 11,667 expressed genes were detected in the porcine liver samples (21.77% of the 53,600 total annotated genes for pig in the UniGene transcriptome assembly).

### Detection and characterization of nsSNV candidates in porcine liver transcriptome reads

**Figure 1:**
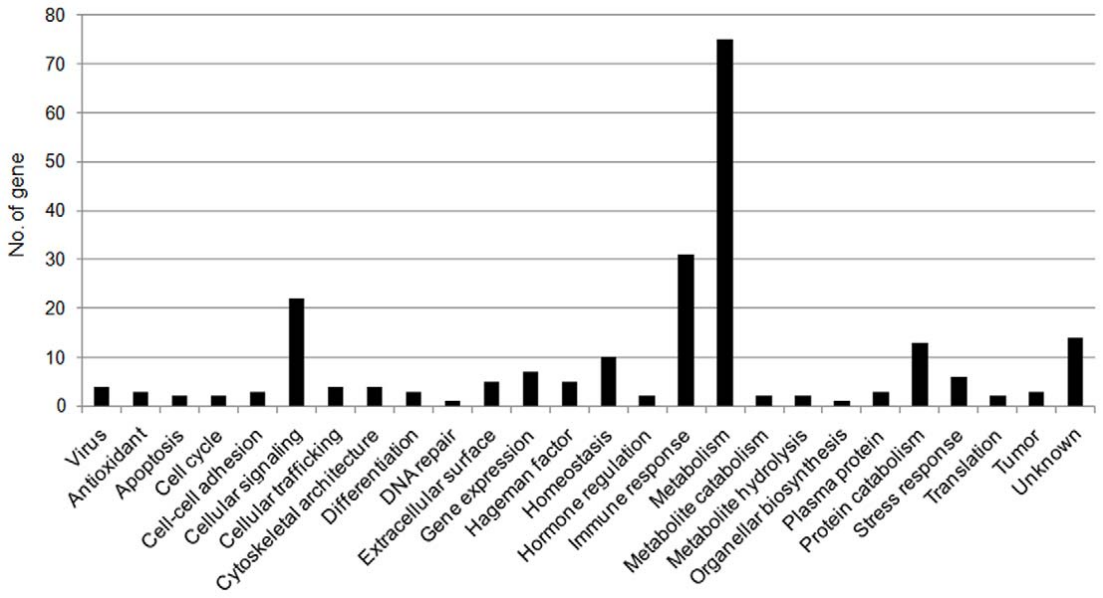
Functional classification of genes containing nonsynonymous SNV candidates. A total of 229 genes were found to include the 580 nonsynonymous SNV candidates shown in Table 1. In addition, they were further classified according to function. The vertical axis on the graph (“No. of gene”) represents the total number of genes.

Subsequently, the mapped reads were subjected to transcriptomic SNV discovery in four pig breeds, which was undertaken on the basis of exon reads showing nucleotide variation rates of greater than 30% among genes with a coverage of more than 30 reads. Using this strategy, 18,969 SNV candidates were identified in 4,248 genes, but location information in the gene sequence was available for only 3,623 of these candidates. This information was obtained using the NCBI database, with searches based on the start codons of these gene coding sequences (Table S1 and Table 1); the UTRs contained 2,168 (5′-UTR, 35; 3′-UTR, 2,133) SNV candidates and the ORFs contained 1,455 SNV ones. Noticeably, of the 1,455 candidates in the ORFs, 580 ones were nonsynonymous (Table S2). When genes including the 580 nsSNV ones were further classified into various functional categories, many metabolism- or immune-related genes were found to contain nsSNV candidates (Fig. 1), a reasonable result for RNAs extracted from liver tissues.

**Table 1:** Summary of SNV candidates obtained by RNA-seq.

alocation of SNV candidates	No. of SNV candidates (%)
5′-UTR	35 (1.0)
3′-UTR	2,133 (58.9)
bORF	
Synonymous SNVs	875 (24.1)
Non-synonymous SNVs	580 (16.0)
Total identified SNV candidates	3623 (100)
cNon-identified SNV candidates	15,346
dTotal SNV candidates	18,969

a SNV candidates were identified according to information in the UniGene database.

b ORF: open reading frame.

c Non-identified SNVs: SNV candidates that have not been identified in the UniGene database.

d Total SNV candidates: the sum of identified and non-identified SNV candidates.

### Validation of nsSNV candidates detected by porcine liver RNA-Seq

To confirm the presence of different alleles in the nsSNV loci found by RNA-Seq, 45 of the nsSNV candidates for which genomic locations in the pig genome assembly (SGSC Sscrofa9.2/susScr2) were known were selected and genotyped. First, a total of 437 pigs of the pure *Berkshire* line that had been bred under the same conditions, were selected randomly and slaughtered in 10 batches when their body weight reached 110 kg. Subsequently, genomic DNA was isolated from whole blood cells and subjected to genotyping analysis using the Illumina VeraCode GoldenGate system together with BeadStudio software (Illumina) which was used for genotype clustering and calling. As shown in Table 2, this genotyping revealed high call rates (>90%) and more than 0.01 minor allele frequencies (MAFs) at 27 SNV candidates. In addition, the genotype distributions for 33 SNV candidates in this sample population were in agreement with Hardy–Weinberg equilibrium (HWE) (*P*>0.05).

**Table 2:** List of nsSNVs validated in porcine liver genes using RNA-Seq and a genotyping assay.

Gene description	GenBank Acc. No.	aSNV location	Allele variation	bNon-syn	cMAF	dHWE	Call rate (%)
*Sus scrofa* plasminogen	DQ530369	ch1:8099615	T/C	Y>H	0.002	0.96	98.86
		ch1:8115591	C/T	H>Y	0	-	100
		ch1:8120372	A/G	I>V	0.002	0.96	98.40
Solute carrier family 7 member 2	EU155140	ch17:5033342	G/A	V>I	0.304	0.22	63.62
Medium-chain acyl-coA dehydrogenase	AY705916	ch6:96131298	G/A	R>Q	0.115	0.02	81.92
Pituitary tumor-transforming protein	AF339886	ch16:60578202	G/A	A>T	0.001	0.98	99.77
Flavin-containing monooxygenase	M32031	ch9:61645860	T/A	L>I	0.321	<0.001	55.78
Paraoxonase 3 transcript variant 1	EF537044	ch9:70258855	A/G	H>R	0.414	0.66	99.31
Multi-drug resistance associated protein 2	DQ530510	ch14:115791345	C/T	T>M	0.421	0.02	98.86
1-Acylglycerol-3-phosphate O-acyltransferase 5	FJ439668	ch15:34951693	A/G	I>V	0.206	0.21	98.40
*Sus scrofa* hepatic lipase	FJ436379	ch1:117474006	G/A	A>T	0.001	0.98	99.54
		ch1:117474991	G/C	G>R	0.166	<0.001	99.54
Hydroxysteroid (17-beta) dehydrogenase 4	X78201	ch2:111808336	A/G	N>S	0.008	0.86	96.34
		ch2:111829092	A/G	I>V	0.399	0.84	86.27
Microsomal epoxide hydrolase 1	AB000883	ch10:13968962	T/G	S>A	0.419	<0.001	95.19
		ch10:13969569	C/T	T>I	0.207	0.64	99.54
Signal transducer and transcription activator	AB004061	ch5:20728297	A/G	Q>R	0.076	0.31	99.54
Aminolevulinate delta-synthase 1	FJ548763	ch13:28811376	G/A	G>S	0.028	0.55	99.77
Complement component 2	AY349422	ch7:27855203	G/A	D>N	0.265	0.01	98.86
Membrane-bound folate binding protein	AF137374	ch9:6264247	A/T	N>I	0.499	<0.001	99.77
Electron transfer flavoprotein α subunit	AY374469	ch7:61651548	G/A	S>N	0.090	0.86	92.68
Peroxisomal D3,D2-enoyl-CoA isomerase	DQ291159	ch7:2302809	C/G	T>S	0.485	0.08	98.40
Hyaluronidase	U14751	ch9:2598399	A/G	N>D	0.010	0.84	90.85
		ch9:2600729	C/A	T>K	0.028	<0.001	98.17
		ch9:2601188	G/A	R>H	0.088	0.41	100
Complement component C9	DQ333198	ch16:21885422	G/T	K>N	0.095	0.34	92.68
		ch16:21889415	A/T	S>C	0.002	0.96	100
		ch16:21895831	A/T	M>L	0.002	<0.001	99.77
Lipid droplet binding protein (CGI-58)	AY902463	ch13:22924695	A/C	N>H	0.101	0.1	90.85
Ribophorin I	AJ293582	ch13:59221591	G/C	V>L	0.041	0.38	94.74
Prothrombin	DQ530370	ch2:13972118	A/T	Y>F	0.264	0.52	90.16
Proline/arginine-rich antibacterial peptides	X75438	ch13:26044904	G/A	R>Q	0.002	<0.001	97.25
Long-chain enoyl-CoA hydratase:3-hydroxyacyl-CoA dehydrogenase precursor	AF028609	ch3:105304083	G/A	R>K	0.005	0.92	100
Cysteine dioxygenase, type I	AB529450	ch2:108676311	G/A	A>T	0.133	0.05	100
Cytochrome P450 hydroxylase	Y16417	ch5:3026394	T/C	V>A	0.350	0.58	94.51
		ch5:3026507	T/C	C>R	0.378	0.58	100
Mitochondrial 2,4-dienoyl-CoA reductase	AJ301324	ch4:47922794	G/C	V>L	0.196	0.09	98.86
Histone H1-3-like protein	AY489289	ch7:22112616	A/G	T>A	0.353	0.99	99.08
Jumping translocation breakpoint	EU616815	ch4:99943976	C/T	A>V	0.039	0.40	99.31
Bromodomain-containing protein 2	EU402599	ch7:29535980	G/C	G>A	0.171	0.44	84.90
Fructose kidney cortex 1,6-bisphosphatase	M86347	ch10:26859669	A/G	M>V	0.001	0.98	95.88
Iodotyrosine dehalogenase 1	AY426609	ch1:16569215	G/T	M>I	0.001	0.98	99.77
Corticosteroid binding globulin precursor	AF324155	ch7:123934125	G/A	G>R	0.252	<0.001	91.99
Glutathione S-transferase	Z69585	ch7:135748203	A/T	Y>F	0.002	0.96	100
Glutathione peroxidase 3	AY368622	ch16:69410557	A/C	E>A	0.377	0.61	98.86
Porcine testicular carbonyl reductase	M80709	-	G/T	E>stop	0	-	100

nsSNVs, identified by RNA-Seq, were validated by Illumina VeraCode GoldenGate genotyping.

a SNV location is based on the pig genome assembly (SGSC Sscrofa9.2/susScr2).

b Nonsyn represents nonsynonymous variation, leading to the change of an amino acid.

c,d Minor allele frequency and χ^2^-test *p* value for Hardy–Weinberg equilibrium, respectively.

### Discovery of nsSNVs associated with multiple pork meat quality traits

Among the nsSNVs validated above, 18 nsSNVs showing high call rates (>90%) and more than 0.01 MAFs, the genotype distributions of which were in HWE (*p*>0.05), were further subjected to association analysis with pork meat quality traits. For this, meat samples from the 437 pigs in Table 2 were used for meat quality evaluation. Meat quality traits such as backfat thickness, carcass weight, meat color, drip loss, cooking loss, shear force, water holding capacity, post-mortem pH, and chemical compositions (fat, protein, collagen, and moisture) were collected for statistical analysis, as described in the ‘Materials and Methods’ section. As shown in Table S3, 15 nsSNVs were significantly associated (*p*<0.01 or *p*<0.05) with meat quality traits under the codominant model. Especially, 10 nsSNVs showed significant associations with more than three kinds of traits (Table 3). In particular, *ch7:g.22112616A>G* SNV was associated significantly with multiple meat quality traits, such as backfat thickness, meat color (yellowness), drip loss, shear force, water holding capacity and postmortem pH_24 hr_ under the codominant model and was found in the gene encoding the histone H1.3-like protein, a component of chromatin. The porcine H1.3-like protein is highly homologous to human H1.3 (Acc. No. NP_005311.1) with a sequence identity of about 92.3%. Human H1.3 is reported to contain post–translational modification sites, such as phosphorylation, acetylation, methylation, and ubiquitination sites, which are responsible for the epigenetic regulation of gene expression [24], [25]. The *ch7:g.22112616A>G* SNV creates a single amino acid change (Thr/Ala) at the 145^th^ residue of porcine H1.3-like. This residue is very close to the putative 147^th^ threonine phosphorylation site [26]. Thus, our data suggest that *ch7:g.22112616A>G* SNV affects multiple meat quality traits by affecting the epigenetic regulation of postmortem metabolism-related gene expression. This hypothesis is further supported by previous studies showing that epigenetic transcriptional regulation by chromatin modification has broad effects on economic traits in pig [27], [28], [29]; for example, a *C1354T* SNV site in the *KIAA1717* gene, encoding an H3-K4–specific methyltransferase, showed significant associations with meat quality traits [29]. Taken collectively, our data show that porcine liver RNA-Seq is a useful approach for the selective discovery of nsSNVs associated with meat quality traits, although subsequent integrative approaches are necessary to gain structural and evolutionary insights into the precise associations of nsSNVs with meat quality traits.

**Table 3:** Summary of the significant associations between nsSNVs and meat quality traits.

bSNV location	aMeat quality traits														
	CW	BFT	Meat color			CL	DL	Chemical composition				SF	WHC	Postmortem pH	
			CIE *L*	CIE *a*	CIE *b*			Pro	Fat	Coll	Moi			24 hr	45 min
ch9:70258855	○		▵			▵						▵		○	
ch15:34951693						▵									
ch10:13969569							○	▵						▵	
ch7:61651548			▵				○						○	▵	
ch7:2302809											▵			▵	
ch16:21885422	▵		▵					▵		○	▵			▵	
ch13:22924695		▵				○									▵
ch13:59221591								▵							
ch2:13972118					▵						▵				
ch2:108676311			▵	○						▵					
ch5:3026394							▵					▵	▵		
ch5:3026507							▵	▵	▵						
ch7:22112616		○			○		○					▵	○	○	
ch4:99943976		▵								▵					
ch16:69410557			▵		▵	○									

a Meat quality traits include carcass weight (CW), backfat thickness (BFT), meat color (lightness, CIE *L*; redness, CIE *a*; yellowness, CIE *b*), cooking loss (CW), drip loss (DL), chemical compositions (protein, Pro; fat, Fat; collagen, Coll; moisture, Moi), shear force (SF), water holding capacity (WHC), and postmortem pH (pH_24 hr_; pH_45 min_).

b SNV location is based on the pig genome assembly (SGSC Sscrofa9.2/susScr2). The triangle (▵) and circle (○) represent significant differences (▵, p<0.05; ○, p<0.01) in genotypes under a codominant model.

### Identification of a duplicated gene nucleotide variation that result in premature stop codons

The nsSNVs may be either missense or nonsense, which results in a single amino acid replacement or a premature stop codon, respectively. Particularly, the nonsense variation can alter the stability and function of proteins by leading to the truncation of an amino acid chain and thus can cause some genetic disorders including human diseases [30], [31], [32], [33], [34].

**Figure 2:**
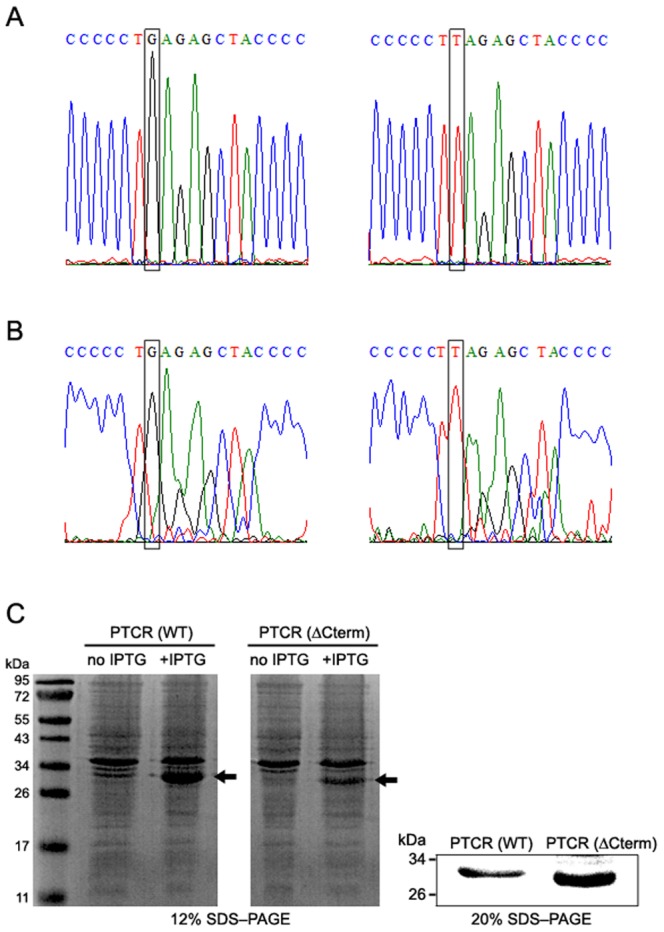
Confirmation of the nonsense variation at the genomic, transcript, and protein levels for *PTCR*. The partial genomic DNA (A) and full length cDNA (B) of *PTCR*, including the nonsense variation locus, were obtained by PCR using specific primers as described in the ‘Methods’ section and subcloned into the pGEM T easy vector for Sanger sequencing. Boxes indicate different variants (G/T) in an exonic region of genome (A) and at nucleotide 951 of the transcript (B). Expression of *PTCR* genes with different variants (G/T) was induced in *E. coli* BL21 by IPTG, and total extracts were loaded onto 12% and 20% SDS-polyacrylamide gels (C). Arrows indicate the His-tagged PTCR fusion proteins of different sizes, about 32 kD or 31 kD, which correspond to PTCR(WT) and PTCR(ΔCterm), respectively.

Noticeably, several genes were identified that included a stop codon caused by a nonsynonymous single nucleotide change. As shown in Table 4, the stop codons were discovered by RNA-Seq analysis in four genes encoding porcine testicular carbonyl reductase 1 (PTCR), type III receptor tyrosine kinase, mannose receptor C type 1 and aldo-keto reductase family 1 C1-like, where they probably caused C-terminal truncations. We focused on PTCR and further validated the nonsense SNV locus in *PTCR* by performing both genotyping for the pig population and Sanger sequencing of its partial genomic DNA and full length cDNA, including the variation site. The genotyping showed only G type, no allelic variation, at the locus of *PTCR* gene in the population (Table 2). However, the sequencing of T-vector-cloned PCR fragments revealed that the gene has different base type (G/T) in an exonic region (Fig. 2A) and, in particular, at the 951^st^ nucleotide of the *PTCR* transcript (Fig. 2B). The different base type (G/T) is likely to be generated by gene duplication during evolution, and thus, it is considered as the paralogous sequence variation, especially a duplicated gene nucleotide variation (DNV) [35].

**Table 4:** Characteristics of nonsense variations resulting in a premature stop codon.

UniGene ID		Ssc.55707	Ssc.15999	Ssc.9229	Ssc.41041
Gene name		Porcine testicular carbonyl reductase 1 (PTCR)	Type III receptor tyrosine kinase	Mannose receptor C type 1	Aldo-keto reductase family 1 member C1-like
aCDS	Start	108	1	1	1
	End	977	1948	4258	1014
	Length	1230	2031	4949	1323
Reference seq.	Seq.	G	G	C	C
	Position	951	1111	3238	958
Variant seq.^b^(variant read no./total read no.)	B	T (29/32)	-	-	-
	D	T (102/102)	-	-	T (271/272)
	Y	-	-	T (45/45)	T (425/427)
	L	T (40/40)	T (48/48)	T (21/63)	T (429/429)
cNon-Syn/Syn	B	Nonsyn E>*	Syn G	Syn R	Syn R
	D	Nonsyn E>*	Syn G	Syn R	Nonsyn R>*
	Y	Syn E	Syn G	Nonsyn R>*	Nonsyn R>*
	L	Nonsyn E>*	Nonsyn G>*	Nonsyn R>*	Nonsyn R>*

a CDS indicates a cDNA, whose start and end positions and length were represented here.

b Among the total RNA-seq reads for a given mRNA (total read No.), the number of variant RNA-seq reads was calculated (variant read No.).

c Non-Syn and Syn indicate non-synonymous and synonymous variations, respectively. The asterisk (*) represents a stop signal encoded by a stop codon. B, D, Y and L indicate *Berkshire*, *Duroc*, *Landrace*, *Yorkshire* breeds, respectively.

The DNV base (T) is expected to create a nonsynonymous single base variation (GAG(Glu)→TAG(stop)) in the genomic and transcript regions of *PTCR* that would lead to a deletion of the C-terminal region from E281 to A288. To further confirm the C-terminal deletion, each of the *PTCR* genes with a different DNV (G/T) was cloned into a plasmid vector for the production of his-tagged (about 1 kD) fusion protein and expressed in *E. coli* BL21. After IPTG induction, SDS-PAGE revealed that the *PTCR* gene possessing the nonsynonymous single base variation (G>T) was expressed as a protein with a size (about 31 kDa) smaller than that (about 32 kD) of wild-type PTCR (Fig. 2C). This is very consistent with a result in previous report [36]; PTCR/20B-HSD proteins were identified as two bands, 31 kDa and 30 kDa proteins from porcine testis, through western blot analysis, but the reason for detection of a minor 30 kDa protein remains unclear [36]. Thus, our results strongly support that the minor 30 kDa protein, detected in porcine testis, is a C-terminal-deleted PTCR, expressed from the *PTCR* gene having a paralogous sequence variant (T), probably generated by gene duplication. Taken together, our data suggest that the nonsynonymous variation (G>T), generated by gene duplication, leads to a deletion of the C-terminal region (E281 to A288) of PTCR, strongly indicating that the pig testicular genome endogenously produces both wild-type and C-terminal-deleted PTCR, 31 kDa and 30 kDa proteins, respectively.

### Molecular docking study of wild-type and C-terminal-deleted PTCRs and human carbonyl reductase

To understand the structural change caused by nonsense single nucleotide variation, we focused on PTCR for three reasons. First, PTCR is useful for the study of active sites that contribute to substrate specificity because of its capacity to bind a wide range of substrates, such as androgens, progestins, prostaglandins, and even a large number of xenobiotics [23]. Second, because its crystal structure has been resolved at high resolution [37], PTCR provides a fine reference structure from which simulations can be generated for subsequent molecular docking studies. Third, in the PTCR structure, the C-terminal fragment from E281 to A288 is reported to be in the vicinity of the active site [37]. This fragment is absent in human carbonyl reductase despite the high homology (about 85%) between PTCR and human carbonyl reductase [23], [37], which suggests that the PTCR-unique C terminus might provide a clue to the differential substrate specificity between porcine and human carbonyl reductases [23].

**Figure 3:**
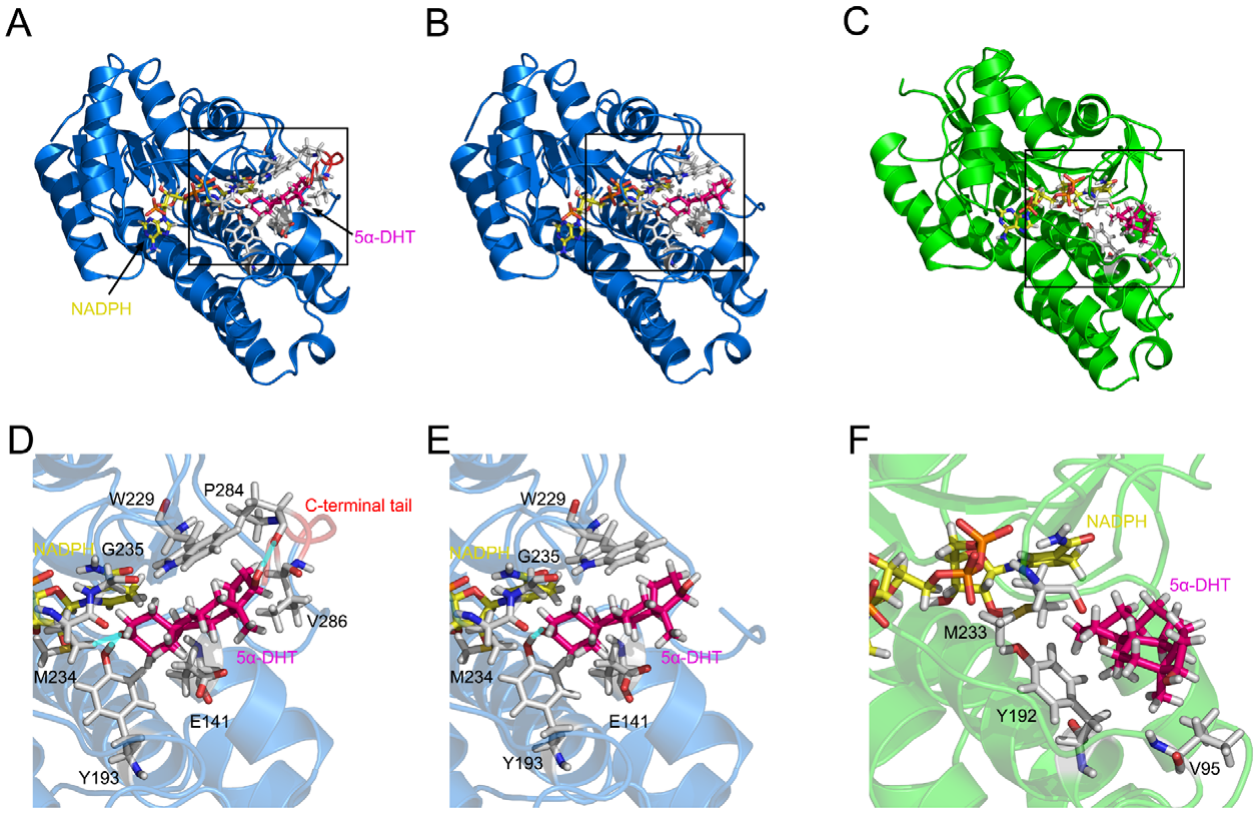
Comparison of the 5α-DHT binding mode among wild-type and C-terminal-deleted PTCRs and human carbonyl reductase. (A) The final conformation of wild-type PTCR (blue, PDB ID: 1N5D) bound with NADPH (yellow) and 5α-DHT substrate (dark pink). The structure contains a C-terminal tail (red, E281-A288). (B) The final conformation of C-terminal-deleted PTCR docked with 5α-DHT. (C) The final conformation of human carbonyl reductase (green, PDB ID: 1WMA) docked with 5α-DHT. (D) Detailed binding mode of 5α-DHT with wild-type PTCR is highlighted by a box in panel (A). Hydrogen bond interactions are represented by blue lines. (E) Detailed binding mode of 5α-DHT with C-terminal-deleted PTCR is highlighted by a box in panel (B). (F) Detailed binding mode of 5α-DHT with human carbonyl reductase is highlighted by a box in panel (C).

Accordingly, molecular docking simulations using 5α-dihydrotestosterone (DHT), a C_21_-steroid, were used to correlate differences in substrate binding between wild-type pork PTCR, C-terminal (E281 to A288)-deleted PTCRs, and human carbonyl reductase with differences in their structures. For the molecular docking study, the 2.30 Å crystal structures of PTCR (PDB ID: 1N5D) and human carbonyl reductase (PDB ID: 1WMA) bound with NADPH were used. PTCR and human carbonyl reductase have both been reported to contain the Tyr-Lys-Ser catalytic triad (S139, Y193, and K197) at their active sites, which is involved in the transfer of hydrogen from NADPH to the substrate [37], [38], [39]. The best-docked conformations were selected according to fitness score and the closest distance between the substrate carbonyl group and the Y193 hydroxyl group that is proposed to be the proton donor for electrophilic attack reactions [39], [40]. The refined conformations of wild-type and C-terminal-deleted PTCRs and human carbonyl reductase were obtained using energy minimization calculations (Fig. 3). In wild-type PTCR, the carbonyl group of 5α-DHT forms two hydrogen bonds with the Y193 hydroxyl group and the M234 sulfur atom. However, in the C-terminal-deleted PTCR, only one hydrogen bond between the carbonyl group and the Y193 hydroxyl group was observed, and human carbonyl reductase showed no hydrogen bond between these residues. In addition, P284, a C-terminal tail (E281-A288) residue, forms hydrogen bond interactions with the hydroxyl group of 5α-DHT in wild-type PTCR. Although both of the PTCR complexes (but not human carbonyl reductase) have similar hydrophobic interactions and identical hydrogen bond interactions between the carbonyl group of 5α-DHT and the Y193 hydroxyl group, stronger charged interactions with 5α-DHT were observed in the wild-type PTCR compared with the C-terminal-deleted PTCR (Table 5). These results suggest that the differences in the expression pattern between wild-type and C-terminal-deleted PTCRs are caused by interaction differences, especially with respect to hydrogen bond interactions between the backbone oxygen atom of P284 and the hydroxyl group of 5α-DHT. This comparative structural analysis of the three complexes revealed stronger hydrogen bond interactions in wild-type PTCR than in C-terminal-deleted PTCR and human carbonyl reductase. Furthermore, our simulation results are strongly supported by the previous report that the deletion of 12 C-terminal residues, including the fragment from E281 to A288, affects steroid metabolism [41]: kinetic comparisons between wild-type and C-terminal-deleted PTCRs revealed that the deletion led to the reduced binding affinity with various steroids, including 5α-dihydrotestosterone (DHT), testosterone and progesterone, resulting in the low enzyme efficiency of PTCR for steroids [41]. Based on these, it can be concluded that wild-type PTCR may have better substrate (5α-DHT) binding affinity than C-terminal-deleted PTCR and human carbonyl reductase.

**Table 5:** Hydrophobic and hydrogen bond interaction profiles.

Systems	Protein-ligand interactions		Binding energy (kcal/mol)
	Hydrogen bonds	Hydrophobic contacts	
WT PTCR	Y193, M234, P284	E141, W229, G235, V286	139.56
C-terminal-deleted PTCR	Y193	E141, W229, M234, G235	148.65
Human carbonyl reductase	-	V96, Y193, M234	157.56

### Functional and evolutionary insights into the conservation of the C-terminal tail in animal carbonyl reductases

**Figure 4:**
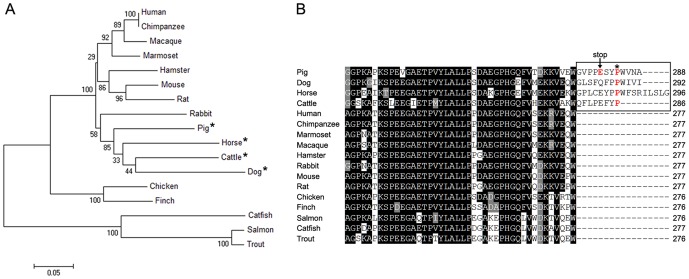
Phylogenetic comparison of PTCR homologs from various organisms. (A) Phylogenetic tree of PTCR homologs. The phylogenetic tree was constructed using the neighbor-joining method and visualized using MEGA4 software. GenBank accession numbers of PTCR homologs are as follows: catfish (ADO28395), cattle (NP_001030258), chicken (NP_001025966), chimpanzee (XP_531449), dog (XP_535589), finch (XP_002187585), hamster (BAB62840), horse (XP_001493595), human (NP_001748), macaque (BAB97216), marmoset (XP_002761453), mouse (NP_031646), pig (NP_999238), rabbit (NP_001076218), rat (NP_062043), salmon (ACI69439), and trout (NP_001118068). Asterisks indicate carbonyl reductases possessing the additional C-terminal tail. (B) Alignment of the amino acid sequences of a C-terminal segment of PTCR homologs in (A). The box indicates additional C-terminal tails. The arrow represents the residue that mutated nonsynonymously into a stop signal in PTCR. The asterisk represents the proline residues conserved among pig, dog, horse, and cattle.

PTCR homologs are conserved in most animals, from fish to human (Fig. 4A). Particularly, PTCR is highly homologous to human carbonyl reductase, with a sequence identity of about 85% [23]. However, PTCR and human carbonyl reductase exhibit a large difference in their substrate specificities; among the compounds used to determine the substrate specificity of human carbonyl reductase, quinones and ketoaldehydes were preferentially reduced, whereas PTCR reduced menadione (one of the quinones) at a lower rate than that of human carbonyl reductase [23], [42]. Steroid hormones such as 5α-DHT are reduced more readily by PTCR than by human carbonyl reductase, suggesting a greater role for PTCR in the reduction of C_21_-steroids than in the carbonyl reduction of a variety of carbonyl compounds [23]. In addition, the deletion of 12 C-terminal residues from PTCR led to the decreased binding affinity with C_21_-steroids, resulting in the low enzyme efficiency of PTCR for the steroids [41]. Moreover, these enzymatic properties of wild-type and C-terminal-deleted PTCRs are strongly supported by the molecular docking simulations which revealed that wild-type PTCR has a higher 5α-DHT-binding affinity than C-terminal-deleted PTCR, similar to human carbonyl reductase. Noticeably, PTCR contains 13 additional amino acid residues at its C terminus, compared with human carbonyl reductase [23], and a similar C-terminal tail was discovered in the carbonyl reductases of animals such as cattle, horse, and dog (Fig. 4B). Sequence alignment also showed that the P284 residue, mediating the hydrogen bond interaction between PTCR and 5α-DHT (Fig. 3 and Table 5), is highly conserved in the C-terminal tails of carbonyl reductase homologs from cattle, horse, and dog (Fig. 4B).

Although further biochemical study will be necessary to confirm whether P284 is indeed a critical site for the 5α-DHT-binding affinity of PTCR, the present study indicates that this residue is evolutionarily conserved and important for the specific binding of PTCR to the steroid hormone 5α-DHT. Therefore, in response to evolutionary pressure to increase substrate specificity for C_21_-steroids, animal carbonyl reductases, including PTCR, may have evolved their C-terminal tails by generating a paralogous sequence variant through gene duplication.

In conclusion, the objective of this study was to selectively discover nonsynonymous single nucleotide variations that are valuable genetic markers for the improvement of economic traits in pig and to highlight the potential use of such variations as a resource for gaining insights into the changes in protein structure that are associated with the gain or loss of protein functions during the curse of evolution. In the present study, porcine metabolism-related genes were identified that included massive nonsynonymous single nucleotide variations. Further, these genes were validated to include different nsSNV alleles with significant associations with various meat quality traits. Moreover, the study shows that nonsynonymous changes may have critical effects on protein structural and functional changes during evolutionary adaptation. For example, a nonsense sequence variation in *PTCR* provided a structural and evolutionary explanation for the specificity of animal carbonyl reductases for an androgen, 5α-DHT. Taken collectively, our integrative approaches may provide useful resources for not only marker-assisted selection in the pig industry but also for protein structure databases to enhance understanding of protein folding, substrate specificity, and the evolution of enzymes.

## Materials and Methods

### Ethics statement

The Animal Care and Use Committee of GNTECH (Gyeongnam National University of Science and Technology) specifically waived the need for consent because no ethics committee approval of study is required for the slaughter of farm animals in the Republic of Korea. However, pigs used in this study were slaughtered in accordance with the guidelines on animal care and use established by the Animal Care and Use Committee of GNTECH and with the Korea Animal Protection Act and related law. In detail, pigs weighing approximately 110 kg were transported to an abattoir near the experimental station. They were slaughtered by stunning with electrical tongs (300 volts for 3 s) after 12 h of feed restriction. The shocked pigs were exsanguinated while being hanged.

### RNA-Seq library preparation

Total RNAs were isolated from liver tissues of the four pigs, consisting of *Berkshire*, *Duroc*, *Landrace,* and *Yorkshire* breeds, using TRI-Reagent (Molecular Research Center, Cincinnati, OH, USA) according to the manufacturer's instructions, and mRNA was isolated and purified using an RNA-Seq sample preparation kit (Illumina, Inc., San Diego, CA). The mRNA was fragmented and used as a template for first- and second-strand cDNA synthesis. After adapters were ligated to the ends of the double-stranded cDNA, a 300±25 bp fragment size was selected by gel excision and each sample was individually sequenced on an Illumina GAII analyzer.

### RNA-Seq analysis and detection of SNV candidates

Using a sliding window method, the reads, obtained by the above sequencing, are further trimmed according to their base qualities: with a specific base sliding window, if the average quality value for this window is higher than a threshold (T = 20), then the window sliding continues, whereas, if it is lower than the threshold, then this stretches of sequence are trimmed out from the original read. Thus, our RNA-Seq finally included a total of 97,524,874 trimmed reads. The trimmed reads were further assembled and mapped to the UniGene, an annotated pig transcriptome assembly (http://www.ncbi.nlm.nih.gov/UniGene/UGOrg.cgi?TAXID = 9823), by performing alignments using BWA software [43]. At the mapping procedure, we allowed up to two mismatches per 32 nucleotides of the trimmed reads. Of these, 52,417,707 reads (∼54%) were categorized as mapped reads, corresponding to exon reads, and the read coverage in exon regions was obtained by BEDtools [44]. In addition, RPKM (reads per kilobase per million mapped reads) values were generated, according to a previous report [45], and were used to identify the total number of genes expressed in the porcine liver. Using RPKM threshold values greater than 1, 11,667 expressed genes were detected in porcine liver samples; 21.77% of the 53,600 total pig annotated genes in the UniGene transcriptome assembly. Finally, the mapped reads were subjected to exonic SNV discovery, which was achieved on the basis of exon reads showing nucleotide variation rates of >30% among genes with greater than 30 read coverage. Synonymous and nonsynonymous SNVs were identified on the basis of NCBI information regarding the start codon, and the functional classification of genes, containing nonsynonymous SNV candidates, was carried out through Gene Ontology and KEGG pathway analyses using DAVID web tool [46]. For running the tool, we used the 11,667 expressed genes as input data and the DAVID gene IDs, based on NCBI Accession IDs, in the porcine background supplied by DAVID. Among functional annotations of the expressed genes, those of the 229 genes, found to include the 580 nonsynonymous SNV candidates (Table 1), were further classified according to functions shown in Fig. 1.

### SNV validation

A total of 437 pigs of pure *Berkshire* line, bred under the same conditions, were selected randomly and slaughtered in 10 batches when their body weight reached 110 kg. Subsequently, genomic DNAs were isolated from individuals' whole blood cells and subjected to SNV genotyping analysis. The Illumina VeraCode GoldenGate Assay kit (Illumina, San Diego, CA) was used to perform the genotyping of nsSNVs, according to the manufacturer's instructions. Primer information for the genotyping of 45 nsSNVs is summarized in Table S4. Genotype clustering and calling were performed using BeadStudio software (Illumina). Meanwhile, the genotyping of an nsSNV in *PTCR* was carried out through the sequencing of PCR products obtained with genomic DNAs used in the above multiplex genotyping. In detail, PCR products for the genomic region including the nsSNV were obtained using the following primers: 5′– CCCAGGGTGGGTGAGAACTGACAT–3′ and 5′–TTATGCATTGACCCAGGG–3′ as forward and reverse primers, respectively. Subsequently, they were subjected to sequencing analysis using Applied Biosystems 3130*xl* DNA sequencer (Applied biosystems, Foster City, CA) to obtain genotype calls.

### Association analysis with meat quality traits

Pork meat quality traits such as backfat thickness, carcass weight, meat color, drip loss, cooking loss, shear force, water-holding capacity, postmortem pH and chemical composition (fat, protein, collagen, and moisture) were evaluated and subjected to statistical analysis, as described previously [12], [47].

To clarify the associations between genotype and meat quality traits for each of the 18 SNVs, statistical analysis was performed with SAS version 9.1.3 (SAS Institute Inc., Cary, NC). Only SNVs showing high call rates (>90%) and MAFs greater than 0.01 and whose genotype distributions were in HWE (*p*>0.05) were subjected to statistical analysis. To verify significant differences (*p*<0.01 and *p*<0.05) between the genotypic frequencies of traits, the Mann-Whitney and Student's t tests and the ANOVA and Kruskal-Wallis tests were used for the dominant and recessive models and the codominant model, respectively.

### Validation of the nonsense variation locus of PTCR at the genomic, transcript and protein levels

To validate the nonsense variation of *PTCR*, classical Sanger sequencing was performed on exonic and transcribed regions, including the nonsense variation, because of the unreliability of *PTCR* genomic information. PCR products for the exonic and transcript regions were obtained using the following primers: 5′–ATCACAGAGGAGGAGCTG–3′ and 5′–TTATGCATTGACCCAGGG–3′ as forward and reverse primers, respectively, for the exonic region, and 5′–ATGTCTTCCAACACTCGAG–3′ and 5′– TTATGCATTGACCCAGGG–3′ as forward and reverse primers, respectively, for the transcript region. Subsequently, they were subcloned into a pGEM-T Easy vector (Promega) for sequencing.

To confirm that the C-terminal deletion resulted from the nonsense variation, each of the *PTCR* genes, having different variants (G/T), was expressed in *E. coli* BL21. For this, two types (G/T) of *PTCR* inserts were prepared by PCR and subsequent *Bam HI* and *Hind III* digestion and were subcloned into the pP_RO_EX HTb vector for the production of his-tagged fusion protein, generating two types of pP_RO_EX HTb-*PTCR* clones. *E. coli* BL21 transformed with each of the clones was subjected to IPTG induction and total extracts were loaded onto 12% and 20% SDS-polyacrylamide gels. Finally, the gels were observed by Coomassie brilliant blue staining, and after excision of bands from the gel and protein digestion, PTCR proteins were identified by MALDI-TOF mass spectrometry.

### Molecular docking simulation and energy minimization calculation

Molecular docking simulations were carried out to investigate the binding mode of 5α-DHT into PTCR (PDB ID: 1N5D) and human carbonyl reductase (PDB ID: 1WMA) using the GOLD 4.1 program (Genetic Optimization for Ligand Docking) from Cambridge Crystallographic Data Center, UK [48]. The program utilizes a genetic algorithm for docking flexible ligands into protein binding sites [49]. The GOLD program uses a genetic algorithm (GA) in combination with scoring functions to predict binding conformations. The number of GA runs was set to 30 iterations on each flexible ligand and, to increase accuracy, early termination was not allowed. The GOLD fitness score was adopted to rank order for proper ligand conformations. The ligand binding site radius was taken as 20 Å around the center of the site between the C-terminal tail and NADPH. All other parameters were maintained by default values. Energy minimization (EM) calculations were performed to refine the docked structures using DS 2.5 (Accelrys Inc., San Diego, USA). The Smart Minimizer EM algorithm was used to relax the conformation and remove any steric overlap that would serve to produce bad contacts from initial structures. The algorithm parameters used were as follows: 10,000 max steps, 0.01 kcal/mol RMS Gradient, and distance-dependent dielectrics for the implicit solvent model.

## Supporting Information

Table S1 **Exonic SNPs obtained by pig liver RNA-Seq analysis.** Y, L, B and D indicate Yorkshire, Landrace, Berkshire and Duroc, respectively. Syn and non-syn represent synonymous and nonsynonymous SNPs, respectively. The * indicates a stop codon.
(XLS)

Table S2 **Summary of 580 nonsynonymous SNV candidates.** Y, L, B and D indicate Yorkshire, Landrace, Berkshire and Duroc, respectively. Syn and non-syn represent synonymous and nonsynonymous SNV candidates, respectively. The * indicates a stop codon.
(XLS)

Table S3 **Association analysis of non-synonymous SNVs with pork meat quality traits.** To verify significant differences (p<0.01 and p<0.05) of traits between genotypic frequencies, the Mann-Whitney and Student's t tests and the ANOVA and Kruskal-Wallis tests were used for the dominant and recessive models and the codominant model, respecively.
(XLS)

Table S4 **Primer information used for the genotyping of 45 nsSNVs.** Three oligonucleotides were designed for the Illumina VeraCode GoldenGate genotyping analysis of each SNV locus, according to the manufacturer's instructions (Illumina, San Diego, CA). For each SNV site there are two allele-specific oligos (ASO). A third oligo, the locus-specific oligo (LSO), hybridizes several bases downstream from the SNV site.
(XLS)

## References

[1] Cherel P, Herault F, Vincent A, Le Roy P, Damon M (2012) Genetic variability of transcript abundance in pig skeletal muscle at slaughter: Relationships with meat quality traits. J Anim Sci 90: 699–708. 21984720 10.2527/jas.2011-4198

[2] Wood J, Enser M, Fisher A, Nute G, Sheard P, et al (2008) Fat deposition, fatty acid composition and meat quality: A review. Meat Sci 78: 343–358. 22062452 10.1016/j.meatsci.2007.07.019

[3] Warner R, Greenwood P, Pethick D, Ferguson D (2010) Genetic and environmental effects on meat quality. Meat Sci 86: 171–183. 20561754 10.1016/j.meatsci.2010.04.042

[4] Ponsuksili S, Murani E, Brand B, Schwerin M, Wimmers K (2011) Integrating expression profiling and whole genome association for dissection of fat traits in a porcine model. J Lipid Res doi: 10.1194/jlr.M013342. 10.1194/jlr.M013342 PMC3284160 21289033

[5] Lalonde E, Ha KCH, Wang Z, Bemmo A, Kleinman CL, et al (2010) RNA sequencing reveals the role of splicing polymorphisms in regulating human gene expression. Genome Res doi: 10.1101/gr.111211.111110. 10.1101/gr.111211.110 PMC3065702 21173033

[6] Shoemaker R, Deng J, Wang W, Zhang K (2010) Allele-specific methylation is prevalent and is contributed by CpG-SNPs in the human genome. Genome Res 20: 883–889. 20418490 10.1101/gr.104695.109 PMC2892089

[7] Zheng W, Zhao H, Mancera E, Steinmetz LM, Snyder M (2010) Genetic analysis of variation in transcription factor binding in yeast. Nature 464: 1187–1191. 20237471 10.1038/nature08934 PMC2941147

[8] Kasowski M, Grubert F, Heffelfinger C, Hariharan M, Asabere A, et al (2010) Variation in transcription factor binding among humans. Science 328: 232–235. 20299548 10.1126/science.1183621 PMC2938768

[9] Wojcik SE, Rossi S, Shimizu M, Nicoloso MS, Cimmino A, et al (2010) Non-codingRNA sequence variations in human chronic lymphocytic leukemia and colorectal cancer. Carcinogenesis 31: 208–215. 19926640 10.1093/carcin/bgp209 PMC2812567

[10] Uzun A, Leslin C, Abyzov A, Ilyin V (2007) Structure SNP (StSNP): a web server for mapping and modeling nsSNPs on protein structures with linkage to metabolic pathways. Nucleic Acids Res 35: W384–W392. 17537826 10.1093/nar/gkm232 PMC1933130

[11] Yang JO, Oh S, Ko G, Park SJ, Kim WY, et al (2011) VnD: a structure-centric database of disease-related SNPs and drugs. Nucleic Acids Res 39: D939–D944. 21051351 10.1093/nar/gkq957 PMC3013797

[12] Fan B, Lkhagvadorj S, Cai W, Young J, Smith R, et al (2010) Identification of genetic markers associated with residual feed intake and meat quality traits in the pig. Meat Sci 84: 645–650. 20374837 10.1016/j.meatsci.2009.10.025

[13] Sunyaev S, Ramensky V, Koch I, Lathe W III, Kondrashov AS, et al (2001) Prediction of deleterious human alleles. Hum Mol Genet 10: 591–597. 11230178 10.1093/hmg/10.6.591

[14] Chasman D, Adams RM (2001) Predicting the functional consequences of non-synonymous single nucleotide polymorphisms: structure-based assessment of amino acid variation1. J Mol Biol 307: 683–706. 11254390 10.1006/jmbi.2001.4510

[15] McCabe M, Waters S, Howard D, Giblin L, Magee D, et al (2010) Discovery of novel single nucleotide polymorphisms in the bovine growth hormone receptor gene and their association with performance traits in Holstein-Friesian cattle in Ireland. Adv Anim Biosci 1: 62–62.

[16] Li Y, Vinckenbosch N, Tian G, Huerta-Sanchez E, Jiang T, et al (2010) Resequencing of 200 human exomes identifies an excess of low-frequency non-synonymous coding variants. Nat Genet 42: 969–972. 20890277 10.1038/ng.680

[17] Eck SH, Benet-Pagès A, Flisikowski K, Meitinger T, Fries R, et al (2009) Whole genome sequencing of a single Bos taurus animal for single nucleotide polymorphism discovery. Genom Biol 10: R82. 10.1186/gb-2009-10-8-r82 PMC2745763 19660108

[18] Burton PR, Clayton DG, Cardon LR, Craddock N, Deloukas P, et al (2007) Association scan of 14,500 nonsynonymous SNPs in four diseases identifies autoimmunity variants. Nat Genet 39: 1329–1337. 17952073 10.1038/ng.2007.17 PMC2680141

[19] Li R, Li Y, Fang X, Yang H, Wang J, et al (2009) SNP detection for massively parallel whole-genome resequencing. Genome Res 19: 1124–1132. 19420381 10.1101/gr.088013.108 PMC2694485

[20] Rubin CJ, Zody MC, Eriksson J, Meadows JRS, Sherwood E, et al (2010) Whole-genome resequencing reveals loci under selection during chicken domestication. Nature 464: 587–591. 20220755 10.1038/nature08832

[21] Chepelev I, Wei G, Tang Q, Zhao K (2009) Detection of single nucleotide variations in expressed exons of the human genome using RNA-Seq. Nucleic acids Res 37: e106. 19528076 10.1093/nar/gkp507 PMC2760790

[22] Canovas A, Rincon G, Islas-Trejo A, Wickramasinghe S, Medrano J (2010) SNP discovery in the bovine milk transcriptome using RNA-Seq technology. Mamm Genome 21: 592–598. 21057797 10.1007/s00335-010-9297-z PMC3002166

[23] Tanaka M, Ohno S, Adachi S, Nakajin S, Shinoda M, et al (1992) Pig testicular 20 beta-hydroxysteroid dehydrogenase exhibits carbonyl reductase-like structure and activity. cDNA cloning of pig testicular 20 beta-hydroxysteroid dehydrogenase. J Biol Chem 267: 13451–11345. 1377683

[24] Wisniewski JR, Zougman A, Kruger S, Mann M (2007) Mass spectrometric mapping of linker histone H1 variants reveals multiple acetylations, methylations, and phosphorylation as well as differences between cell culture and tissue. Mol Cell Proteomics 6: 72–87. 17043054 10.1074/mcp.M600255-MCP200

[25] Margueron R, Reinberg D (2010) Chromatin structure and the inheritance of epigenetic information. Nat Rev Genet 11: 285–296. 20300089 10.1038/nrg2752 PMC3760772

[26] Garcia BA, Busby SA, Barber CM, Shabanowitz J, Allis CD, et al (2004) Characterization of phosphorylation sites on histone H1 isoforms by tandem mass spectrometry. J Proteome Res 3: 1219–1227. 15595731 10.1021/pr0498887

[27] Andersson L, Georges M (2004) Domestic-animal genomics: deciphering the genetics of complex traits. Nat Rev Genet 5: 202–212. 14970822 10.1038/nrg1294

[28] Peng Y, Yerle M, Liu B (2009) Mapping and expression analyses during porcine foetal muscle development of 12 genes involved in histone modifications. AnimGenet 40: 242–246. 10.1111/j.1365-2052.2008.01818.x 19133938

[29] Xu D, Liu M, Xiong Y, Deng C, Jiang S, et al (2007) Identification of polymorphisms and association analysis with meat quality traits in the porcine KIAA1717 and HUMMLC2B genes. Livestock Sci 106: 96–101.

[30] Chai LYA, De Boer MGJ, Van Der Velden WJFM, Plantinga TS, Van Spriel AB, et al (2011) The Y238X stop codon polymorphism in the human β-glucan receptor dectin-1 and susceptibility to invasive aspergillosis. J Infect Dis 203: 736–743. 21242599 10.1093/infdis/jiq102 PMC3072717

[31] Sampath V, Garland JS, Le M, Patel AL, Konduri GG, et al (2011) A TLR5 (g. 1174C>T) variant that encodes a stop codon (R392X) is associated with bronchopulmonary dysplasia. Pediatric Pulmonol 47: 460–468. 10.1002/ppul.21568 22058078

[32] Philibert P, Audran F, Pienkowski C, Morange I, Kohler B, et al (2010) Complete androgen insensitivity syndrome is frequently due to premature stop codons in exon 1 of the androgen receptor gene: an international collaborative report of 13 new mutations. Fertil Steril 94: 472–476. 19463997 10.1016/j.fertnstert.2009.03.057

[33] Wu X, Wan S, Pujar S, Haskins ME, Schlafer DH, et al (2009) A single base pair mutation encoding a premature stop codon in the MIS type II receptor is responsible for canine persistent Mullerian duct syndrome. J Androl 30: 46–56. 18723470 10.2164/jandrol.108.005736 PMC2593750

[34] Savas S, Tuzmen S, Ozcelik H (2006) Human SNPs resulting in premature stop codons and protein truncation. Hum Genomics 2: 274–286. 16595072 10.1186/1479-7364-2-5-274 PMC3500177

[35] Ho MR, Tsai KW, Chen C, Lin W (2011) dbDNV: a resource of duplicated gene nucleotide variants in human genome. Nucleic Acids Res 39: D920–D925. 21097891 10.1093/nar/gkq1197 PMC3013738

[36] Kobayashi K, Ohno S, Shinoda M, Toyoshima S, Nakajin S (1996) Immunochemical distribution and immunohistochemical localization of 20 [beta]-hydroxysteroid dehydrogenase in neonatal pig tissues. J Steroid Biochem Mol Biol 59: 485–493. 9010354 10.1016/s0960-0760(96)00137-9

[37] Ghosh D, Sawicki M, Pletnev V, Erman M, Ohno S, et al (2001) Porcine Carbonyl Reductase. J Biol Chem 276: 18457–18463. 11279087 10.1074/jbc.M100538200

[38] Geissler R, Brandt W, Ziegler J (2007) Molecular modeling and site-directed mutagenesis reveal the benzylisoquinoline binding site of the short-chain dehydrogenase/reductase salutaridine reductase. Plant physiol 143: 1493–1503. 17337529 10.1104/pp.106.095166 PMC1851842

[39] Bateman RL, Rauh D, Tavshanjian B, Shokat KM (2008) Human carbonyl reductase 1 is an S-nitrosoglutathione reductase. J Biol Chem 283: 35756–35762. 18826943 10.1074/jbc.M807125200 PMC2602912

[40] Ghosh D, Wawrzak Z, Weeks C, Duax W, Erman M (1994) The refined three-dimensional structure of 3 [alpha], 20 [beta]-hydroxysteroid dehydrogenase and possible roles of the residues conserved in short-chain dehydrogenases. Structure 2: 629–640. 7922040 10.1016/s0969-2126(00)00064-2

[41] Nakajin S, Itoda M, Ohno S, Takase N, Toyoshima S, et al (2001) Deletion of 12 carboxyl-terminal residues from pig 3[alpha]/[beta], 20[beta]-hydroxysteroid dehydrogenase affects steroid metabolism. Biochim Biophys Acta, Protein Struct Mol Enzymol 1550: 175–182. 10.1016/s0167-4838(01)00285-0 11755206

[42] Wermuth B (1981) Purification and properties of an NADPH-dependent carbonyl reductase from human brain. Relationship to prostaglandin 9-ketoreductase and xenobiotic ketone reductase. J Biol Chem 256: 1206–1213. 7005231

[43] Li H, Durbin R (2010) Fast and accurate long-read alignment with Burrows–Wheeler transform. Bioinform 26: 589–595. 10.1093/bioinformatics/btp698 PMC2828108 20080505

[44] Quinlan A, Hall I (2010) BEDTools: a flexible suite of utilities for comparing genomic features. Bioinformatics 26: 841–842. 20110278 10.1093/bioinformatics/btq033 PMC2832824

[45] Mortazavi A, Williams B, McCue K, Schaeffer L, Wold B (2008) Mapping and quantifying mammalian transcriptomes by RNA-Seq. Nature methods 5: 621–628. 18516045 10.1038/nmeth.1226 PMC13303166

[46] Da Wei Huang BTS, Lempicki RA (2008) Systematic and integrative analysis of large gene lists using DAVID bioinformatics resources. Nat Protoc 4: 44–57. 10.1038/nprot.2008.211 19131956

[47] Li X, Yang X, Shan B, Shi J, Xia D, et al (2009) Meat quality is associated with muscle metabolic status but not contractile myofiber type composition in premature pigs. Meat Sci 81: 218–223. 22063985 10.1016/j.meatsci.2008.07.022

[48] Verdonk M, Cole J, Hartshorn M, Murray C, Taylor R (2003) Improved protein-ligand docking using GOLD. Proteins: Structure, Function, and Bioinformatics 52: 609–623. 10.1002/prot.10465 12910460

[49] Jones G, Willett P, Glen R, Leach A, Taylor R (1997) Development and validation of a genetic algorithm for flexible docking1. Journal of Molecular Biology 267: 727–748. 9126849 10.1006/jmbi.1996.0897

